# PNPLA3 I148M Up-Regulates Hedgehog and Yap Signaling in Human Hepatic Stellate Cells

**DOI:** 10.3390/ijms21228711

**Published:** 2020-11-18

**Authors:** Francesca Virginia Bruschi, Matteo Tardelli, Elisa Einwallner, Thierry Claudel, Michael Trauner

**Affiliations:** 1 Hans Popper Laboratory of Molecular Hepatology, Division of Gastroenterology and Hepatology, Department of Internal Medicine III, Medical University of Vienna, 1090 Vienna, Austria; francesca_bruschi@yahoo.it (F.V.B.); mat4005@med.cornell.edu (M.T.); thierry.claudel@meduniwien.ac.at (T.C.); 2 Division of Gastroenterology and Hepatology, Joan and Sanford I. Weill Cornell Department of Medicine, Weill Cornell Medical College, New York, NY 10021, USA; 3 Department of Laboratory Medicine, Center of Translational Research, Medical University of Vienna, 1090 Vienna, Austria; elisa.einwallner@meduniwien.ac.at

**Keywords:** genetic polymorphism, non-alcoholic fatty liver disease, cell metabolism, intracellular signaling

## Abstract

Liver fibrosis represents the wound healing response to sustained hepatic injury with activation of hepatic stellate cells (HSCs). The I148M variant of the *PNPLA3* gene represents a risk factor for development of severe liver fibrosis. Activated HSCs carrying the I148M variant display exacerbated pro-inflammatory and pro-fibrogenic features. We aimed to examine whether the I148M variant may impair Hedgehog and Yap signaling, as key pathways implicated in the control of energy expenditure and maintenance of myofibroblastic traits. First, we show that TGF-β rapidly up-regulated the PNPLA3 transcript and protein and Yap/Hedgehog target gene expression. In addition, HSCs overexpressing *PNPLA3* I148M boosted anaerobic glycolysis, as supported by higher lactate release and decreased phosphorylation of the energy sensor AMPK. These cells displayed higher Yap and Hedgehog signaling, due to accumulation of total Yap protein, Yap promoter activity and increased downstream targets expression, compared to WT cells. HSCs exposed to TGF-β and leptin rapidly increased total Yap, together with a reduction in its inhibited form, phosphorylated Yap. In line, Yap-specific inhibitor Verteporfin strongly abolished Yap-mediated genes expression, at baseline as well as after TGF-β and leptin treatments in HSCs with I148M *PNPLA3*. Finally, Yap transcriptional activity was strongly reduced by a combination of Verteporfin and Rosiglitazone, a PPARγ synthetic agonist. In conclusion, HSCs carrying the *PNPLA3* variant show activated Yap/Hedgehog pathways, resulting in altered anaerobic glycolysis and enhanced synthesis of Hedgehog markers and sustained Yap signaling. TGF-β and leptin exacerbate Yap/Hedgehog-related fibrogenic genes expression, while Yap inhibitors and PPARγ agonists abrogate these effects in *PNPLA3* I148M carrying HSCs.

## 1. Introduction

Due to the rising percentage of global obesity, non-alcoholic fatty liver disease (NAFLD) is estimated to affect approximately 25% of the world’s adult population [1] and become the leading cause of chronic liver disease (CLD). Fibrosis represents a key prognostic determinant for clinical outcomes of CLD including NAFLD [2,3] and is associated with functional organ impairment [1,4], leading to development of end-stage liver disease, cirrhosis and hepatocellular carcinoma (HCC) [5,6]. Sustained liver fibrogenesis results in cirrhosis which is characterized by the loss of normal liver architecture and replacement of wound healing scars, consisting of an altered and abundant extracellular matrix (ECM) deposition. This pathophysiological process is mainly driven by activation of hepatic stellate cells (HSCs) [7], a group of non-parenchymal cells that upon liver injury undergo a phenotypical trans-differentiation towards proliferative activated myofibroblast-like cells [8]. In particular, activated HSCs play an active role in modifying the liver structure and stiffness by the acceleration of hepatocyte growth, migration and increase in the expression of genes involved in epithelial to mesenchymal transition (EMT) [9]. This process is favored by several pleiotropic cytokines, such as the transforming growth factor-beta (TGF-β). TGF-β pro-fibrotic actions are strongly connected to variations in the extracellular matrix (ECM) composition [10], where its pleiotropic actions promote EMT and inhibit cell contacts. TGF-β has been implicated in activation of the Hedgehog pathway (HH) in a non-canonical manner [11] and connected with activation of the Yap/Hippo pathway [12,13]. Interestingly, HH controls features of EMT and reprograms the energy expenditure in HSCs. Moreover, HH was shown to drive HSCs’ fate during activation, since HSCs undergo a metabolic “reprogramming” characterized by accumulation of lactate, due to increased glycolysis and down-regulation of the peroxisome proliferator-activated receptor gamma (PPARγ) [14]. The HH pathway directly modulates the effector of the Hippo pathway, the yes-associated protein 1 (Yap), which has been linked to organ size and liver regeneration, while its alteration promotes several diseases, including fibrosis and cancer [15]. In addition to TGF-β, several mechanisms have been described by which leptin (lep) plays a crucial role in triggering HSC activation, proliferation, maintenance of myofibroblastic traits and EMT during fibrogenesis [16]. As such, leptin stimulates the synthesis of EMT signals and activates HH which translates in the acquisition of mesenchymal features (increased proliferation, induction of pro-fibrotic genes) by activated HSCs to maintain their myofibroblastic features [17].

*PNPLA3*, also known as Adiponutrin, is the most closely related member to *ATGL/PNPLA2* within the PNPLA family, a group of lipid hydrolytic enzymes. A genetic mutation of *PNPLA3*, the I148M polymorphism, has been strongly correlated with a higher risk of developing fibrosis in NAFLD and other liver diseases [18,19]. Recent observations provided a better insight on how PNPLA3 expression is regulated during development of human NASH, highlighting that in early phases, PNPLA3 strongly correlates with α-SMA and is present in α-SMA-positive cells [20]. In addition, molecular analysis showed that lipid- and glucose-stressed hepatocytes induce expression of PNPLA3 specifically in HSCs, thus contributing to trigger a fibrotic response [20]. Notably, HSCs carrying the *PNPLA3* I148M variant were shown to display higher cellular proliferation and migration in concomitance with down-regulation of LXR and PPARγ signaling [21]. Moreover, the latter contributed to enhance endogenous synthesis and release of inflammatory mediators, thus conferring a more pro-inflammatory and pro-fibrogenic phenotype to I148M HSCs [22]. Normally, PPARγ signaling is high when HSCs are in a quiescent state, whereas it is strongly reduced when HSCs acquire myofibroblastic-like tracts [23]. The HH pathway has been reported to down-regulate PPARγ anti-fibrotic actions, since the use of specific inhibitors of HH in activated HSCs re-establishes high PPARγ signaling and reverts activated HSCs to their quiescent phenotype [14].

Given the overlap between HH and Yap signaling and the established actions of TGF-β and leptin on the promotion and maintenance of myofibroblast transformation, we investigated in this study whether TGF-β modulates PNPLA3 expression in HSCs. In addition, we aimed to test if the presence of the *PNPLA3* genetic I148M variant might influence HH/Yap signaling and contribute to the shift in anaerobic metabolism and enhance expression of specific markers which promote myofibroblasts transformation. Finally, we evaluated the effects of TGF-β and leptin on the Yap signaling pathway alone or in combination with Verteporfin, an inhibitor of Yap, in both WT and I148M HSCs. Collectively, our findings expand the suitable range of targets for down-regulating the pro-fibrotic actions of I148M PNPLA3 in HSCs and therefore may contribute to inhibit the onset and progression of liver fibrosis.

## 2. Results

### 2.1. TGF-β Induces PNPLA3 Protein and Transcript Expression and Up-Regulates Yap/Hedgehog Target Genes in HSCs

**Figure 1:**
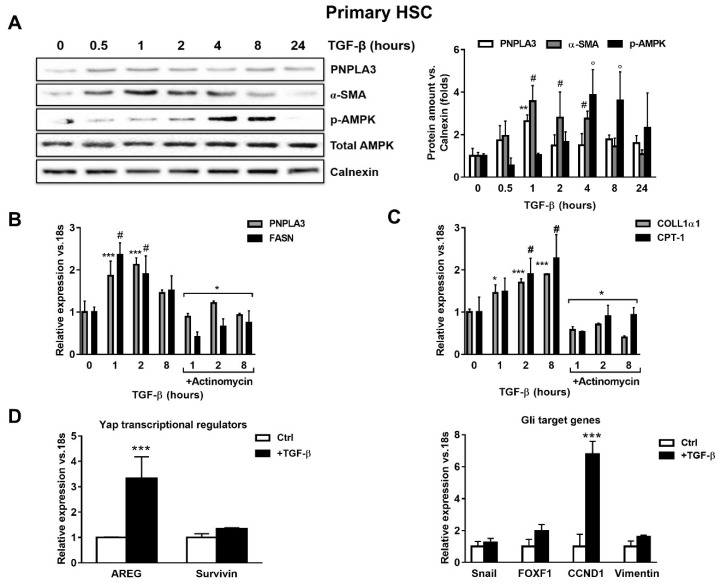
TGF-β increases PNPLA3 and modulates AMPK signaling and Yap/Hedgehog target genes in hepatic stellate cells (HSCs). Primary HSCs (Ctrl) or treated with 5 ng/mL TGF-β at different time points, as indicated in the figure panel. Indicated cells were exposed to Actinomycin for 1h prior TGF-β stimulation. (**A**) Representative blots of PNPLA3, α-SMA, p-AMPK and total AMPK were performed on total protein extracts collected from primary HSCs. Densitometry analysis was calculated using ImageJ software and data were normalized to Calnexin. ** = *p* < 0.01 vs. PNPLA3 without TGF-β (time 0), # = *p* < 0.01 vs. α-SMA without TGF-β (time 0), ° = *p* < 0.001 vs. p-AMPK without TGF-β (time 0). (**B**,**C**) Expression of PNPLA3, FASN COLL1α1 and CPT-1 was analyzed by real-time PCR and normalized to 18s. *** = *p* < 0.001 vs. PNPLA3 without TGF-β (time 0), # = *p* < 0.01 vs. FASN without TGF-β (time 0), * = *p* < 0.001 vs. PNPLA3 and FASN with only TGF-β at different time points (1, 2, 8 h), * = *p* < 0.05, ***= *p* < 0.001 vs. COLL1α1 without TGF-β (time 0), # = *p* < 0.001 vs. CPT-1 without TGF-β (time 0) and * = *p* < 0.001 vs. COLL1α1 and CPT-1 treated with only TGF-β at different time points (1, 2, 8 h). (**D**) Expression of Yap regulators and glioma associated oncogene homolog (GLI) target genes expression: AREG, Survivin, Snail, FOXF1, CCND1 and Vimentin, were analyzed by real-time PCR and normalized to 18s. *** = *p* < 0.001 vs. Ctrl (without TGF-β, white bars). Data displayed represent four independent experiments performed in duplicates. Data shown as mean values ± SD.

To test whether TGF-β modulates PNPLA3 expression in HSCs, we examined by Western blotting PNPLA3 protein levels at different time points after exposure to TGF-β. We found that PNPLA3 was significantly up-regulated after 1 h of TGF-β (*p* < 0.01), along with the HSCs activation marker α-SMA (*p* < 0.01 after 1, 2 and 4 h). Interestingly, the major cellular energy sensor in its active form, phosphorylated 5′ AMP-activated protein kinase (p-AMPK), shows an increased accumulation only at later time points (*p* < 0.001 at 4 and 8 h after TGF-β, Figure 1A). Next, we observed that induction of PNPLA3 by TGF-β was transcriptional, since the RNA-polymerase inhibitor Actinomycin D abolished PNPLA3 mRNA induction (Figure 1B). Importantly, TGF-β induced a time-dependent expression of the representative pro-fibrotic marker Collagen1α1 (COLL1α1) and of carnitine palmitoyl-transferase-1 (CPT-1), whereas the fatty acid synthase (FASN) transcript followed a pattern similar to PNPLA3 (Figure 1B,C).

Next, we examined whether the expression of genes involved in Yap and HH pathways was affected by TGF-β in HSCs. We found that mRNA expressions of Amphiregulin (AREG, *p* < 0.001), Survivin, Snail, forkhead box F1 (FOXF1), Cyclin D1 (CCND1) and Vimentin were all up-regulated after TGF-β treatment (Figure 1D). Collectively, these observations suggest that TGF-β rapidly stimulates expression of PNPLA3 at the mRNA and protein levels in HSCs, accompanied by rapid induction of pro-fibrotic markers (COLL1α1, α-SMA) and FASN, along with Yap/HH downstream targets, whereas longer exposure resulted in activation of the energy sensor AMPK and favored the expression of CPT-1.

### 2.2. HSCs Expressing PNPLA3 I148M Have Enhanced Glycolysis Compared to WT Cells

**Figure 2:**
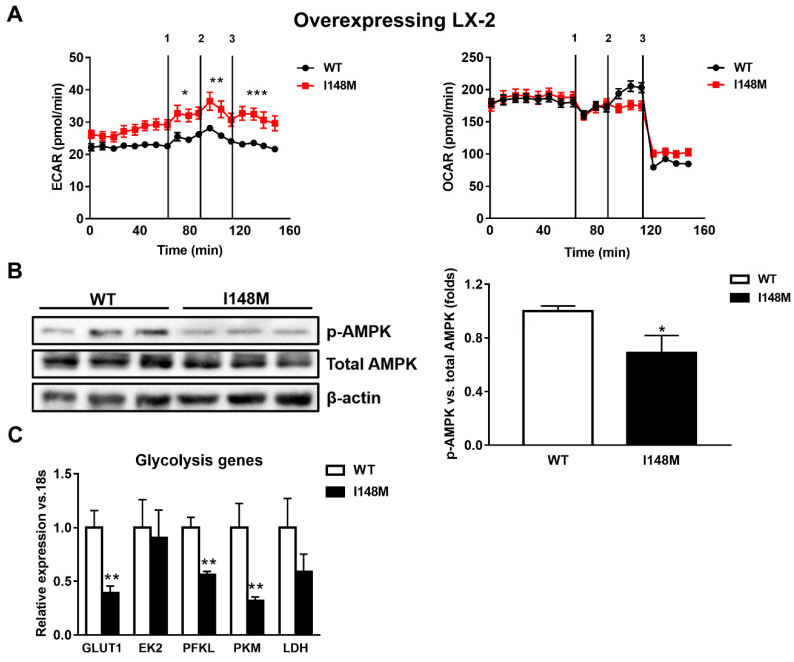
LX-2 with the *PNPLA3* genetic variant shows activated anaerobic glycolysis. LX-2 stably overexpressing cells (*n* = 3 each *PNPLA3* genotype, WT or I148M) was obtained and cultivated in vitro, as described in Materials and Methods. (**A**) Lactate production (extracellular acidification rate (ECAR), pmol/min) and oxygen consumption rates (OCR, pmol/min) were measured in the culture medium, as described in Materials and Methods. * = *p* < 0.05, ** = *p* < 0.01, *** = *p* < 0.001 vs. WT cells. Separated injection of 1 = oligomycin; 2 = FCCP; and 3 = rotenone and antimycin A. (**B**) Representative blots of phospho-AMPK (p-AMPK) were performed on total protein extracts. Densitometry analysis was calculated using ImageJ software and data were normalized to β-actin. * = *p* < 0.05 vs. WT cells. (**C**) Expression of glycolytic genes, such as GLUT1, EK2, PFKL, PFKM and LDH was analyzed by real-time PCR and normalized to 18s. ** = *p* < 0.01 vs. WT cells. Data displayed represent three independent experiments performed in duplicates. Data shown as mean values ± SD.

During myofibroblastic differentiation, HSCs undergo a metabolic reorganization characterized by decreased transcription of de novo lipogenesis-related genes, combined with enhanced expression of glycolytic genes, resulting in elevated glucose catabolism as the major cellular energy supplier [14]. The overall process is one of the consequences of the down-regulation of the nuclear receptor PPARγ. Since PPARγ signaling is reduced in HSCs expressing the *PNPLA3* genetic variant [22], we performed seahorse measurements of the extracellular acidification rate (ECAR) and oxygen consumption rate (OCR) to assess how cellular energy expenditure was altered. Interestingly, LX-2 overexpressing I148M *PNPLA3* accumulated significantly more lactate (ECAR) compared to WT cells (*p* < 0.001, Figure 2A) but we did not observe significant differences in OCR, suggesting a higher anaerobic catabolism in LX-2 with the *PNPLA3* variant. To confirm these observations, we checked the activity of AMPK by measuring its phosphorylated isoform, in both WT and I148M overexpressing LX-2. We found that p-AMPK was significantly decreased in cells carrying the *PNPLA3* I148M variant (*p* < 0.05, Figure 2B). In line, the relative expressions of major genes implicated in the regulation of glycolytic pathways were significantly down-regulated, such as glucose transporter 1 (GLUT1), phosphofructokinase liver type (PFKL) and pyruvate kinase (PK) (*p* < 0.01, Figure 2C). Taken together, these data indicate that HSCs overexpressing I148M *PNPLA3* rely on a higher anabolic energy supply for their metabolism, as shown by enhanced ECAR, thus leading to decreased activation of the energy sensor AMPK and lower transcription of glycolysis regulatory genes.

### 2.3. Hedgehog and Hippo/Yap Signaling are Up-regulated in HSCs with the PNPLA3 I148M Variant

**Figure 3:**
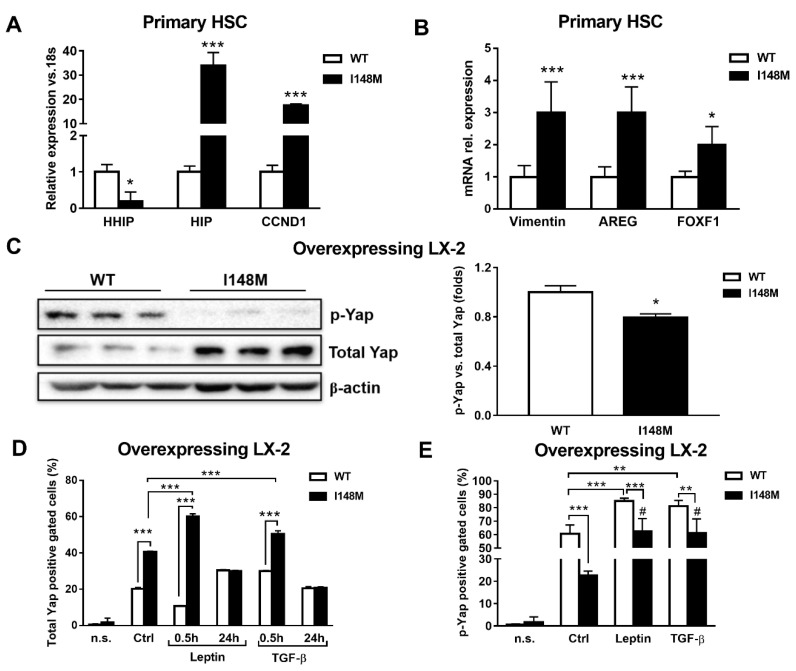
Up-regulated Hedgehog targets result in activation of Yap in HSCs carrying I148M *PNPLA3*. Primary HSCs and LX-2 stably overexpressing cells (*n* = 3 each *PNPLA3* genotype) were cultivated in vitro, as described in Materials and Methods. Where indicated, cells were stimulated with Leptin or TGF-β 1h prior to the analysis. (**A**,**B**) Expression of HHIP, HIP, CCND1, AREG, Vimentin and FOXF1 was analyzed by real-time PCR in primary HSCs carrying WT or I148M *PNPLA3* and normalized to 18s. * = *p* < 0.05, *** = *p* < 0.001 vs. WT HSCs. (**C**) Representative blots of phosphorylated Yap (p-Yap) and total Yap were performed on total protein extracts collected from LX-2 overexpressing WT or I148M *PNPLA3*. Densitometry analysis was calculated using ImageJ software and data were normalized to β-actin. * = *p* < 0.05 vs. WT cells. (**D**) Total Yap intracellular staining was analyzed by flow cytometry in LX-2 overexpressing WT or I148M *PNPLA3*. Data displayed as percentage (%) of gated total Yap-positive cells. *** = *p* < 0.001 vs. the groups indicated in the bar graph. (**E**) p-Yap intracellular staining was analyzed by flow cytometry in LX-2 overexpressing either WT or I148M *PNPLA3*. Data displayed as percentage (%) of gated p-Yap-positive cells. ** = *p* < 0.01, *** = *p* < 0.001 vs. the groups indicated in the bar graph. # = *p* < 0.01 vs. WT cells treated with Leptin or TGF-β. n.s.= not stained cells. Ctrl = untreated cells. Data displayed represent three independent experiments performed in duplicates. Data shown as mean values ± SD.

Yap is a key downstream effector of HH, since disruption of HH signaling blocks both Yap and myofibroblast transformation in HSCs [24,25]. First, we checked whether these two pathways were altered in *PNPLA3* I148M HSCs. Notably, Indian Hedgehog (HIP), cyclin D1 (CCND1), Vimentin, Amphiregulin (AREG) and forkhead box protein F1 (FOXF1) were significantly up-regulated in primary HSCs with the *PNPLA3* variant (*p* < 0.05, *p* < 0.001, Figure 3A), while the transcript of the inhibitor of HH Hedgehog-interacting protein (HHIP) was down-regulated (*p* < 0.05). To confirm these observations, we checked by Western blotting levels of total Yap and its inhibited form, phosphorylated Yap (p-Yap, Ser127), in both WT and I148M overexpressing LX-2. Interestingly, we found that p-Yap was lower in I148M cells compared to WT cells (*p* < 0.05, Figure 3B). To support the Western blotting findings, we performed flow cytometry analysis that confirmed the augmented prevalence of total Yap and lower p-Yap in I148M cells compared to WT counterparts, which was further enhanced by short-term treatment with leptin and TGF-β (at 0.5h *p* < 0.001, Figure 3D). In addition, values of total Yap displayed no significant differences between WT and I148M HSCs after 24 h of treatment with either leptin or TGF-β. These findings on total Yap were supported by the quantity of p-Yap, measured as well by flow cytometry in the same conditions (*p* < 0.001, Figure 3E). Collectively, these data indicate that HSCs carrying I148M display over-activation of HH signaling and its downstream effector Yap compared to WT HSCs. Moreover, Yap accumulation can be further boosted by leptin and TGF-β in HSCs with the *PNPLA3* variant.

### 2.4. The Specific Yap Inhibitor Verteporfin Strongly Reduces Yap-Mediated Gene Expression and TGF-β and Leptin Profibrogenic Actions, in Both WT and I148M Overexpressing HSCs

**Figure 4:**
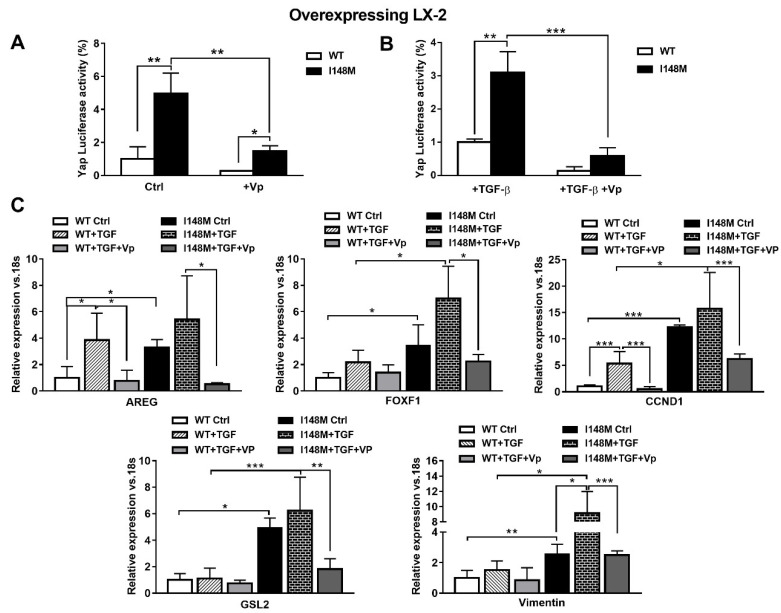
Verteporfin inhibits TGF-β mediated Hedgehog/Yap target gene expression. LX-2 stably overexpressing cells (*n* = 3 each *PNPLA3* genotype) was obtained and cultivated in vitro, as described in Materials and Methods. Where indicated, cells were stimulated with Vp and TGF-β, respectively, for 24 and 1 h prior to the analysis. (**A**) Luciferase activity (%) in HSCs transiently transfected with Yap-promoter luciferase construct untreated (Ctrl) or treated with Vp. * = *p* < 0.05, **= *p* < 0.01 as indicated in the graph. (**B**) Luciferase activity in HSCs transiently transfected with Yap luciferase construct and treated with only TGF-β or with combination of TGF-β + Vp. ** = *p* < 0.01, *** = *p* < 0.001 as indicated in the graph. (**C**) Expression of AREG, FOXF1, CCND1, GLS-2 and Vimentin was analyzed by real-time PCR and normalized to 18s. * = *p* < 0.05, ** = *p* < 0.01, *** = *p* < 0.001 between groups as indicated in the graph. Data displayed represent three independent experiments performed in duplicates. Data shown as mean values ± SD.

TGF-β and leptin have both been implicated in the activation of HH in a non-canonical manner [11,17]. Since Yap is a downstream effector of the HH pathway, we aimed to test whether higher total Yap levels in I148M HSCs translated effectively to higher Yap-mediated signaling. Therefore, we measured Yap transcriptional activity by transient transfection using a luciferase reporter construct carrying a Yap response element in multi-copies linked to a luciferase reporter gene, in WT and I148M overexpressing LX-2. Notably, cells with the *PNPLA3* genetic variant showed a significant difference in Yap transcriptional activity compared to WT HSCs, both at baseline (*p* < 0.01 Figure 4A) and after stimulation with TGF-β (*p* < 0.01 Figure 4B). To confirm that these findings were related to enhanced Yap transcriptional activity, we incubated/treated cells with the specific Yap inhibitor Verteporfin (Vp), prior to measuring luciferase values. Notably, Vp significantly inhibited the Yap-mediated transactivation of its promoter (*p* < 0.01, Figure 4A), also after TGF-β treatment (*p* < 0.001, Figure 4B), thus supporting our hypothesis of increased Yap signaling in I148M *PNPLA3* cells. In line, Vp decreased mRNA expression of HH-Yap downstream targets such as AREG, FOXF1, CCND1, GLS-2 and Vimentin, counteracting stimulation by TGF-β (Figure 4C).

**Figure 5:**
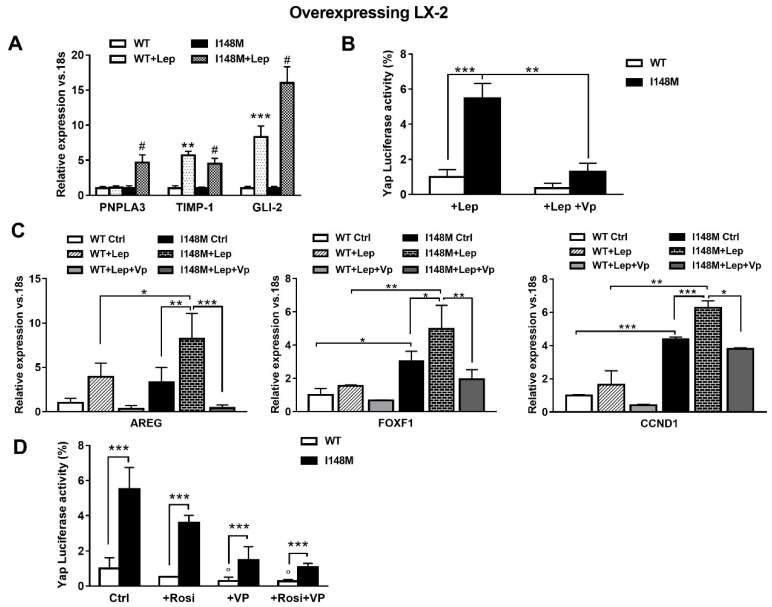
Verteporfin down-regulates leptin-mediated Hedgehog/Yap target gene expression. LX-2 stably overexpressing cells (*n* = 3 each *PNPLA3* genotype) was obtained and cultivated in vitro, as described in Materials and Methods. Where indicated, cells were stimulated with Vp and Lep, respectively, for 24 and 1 h prior to the analysis. (**A**) Expression of PNPLA3, TIMP-1 and GLI-2 was analyzed by real-time PCR and normalized to 18s. ** = *p* < 0.01, *** = *p* < 0.001 vs. WT; # = *p* < 0.001 vs. I148M. (**B**) Luciferase activity (%) in HSCs transiently transfected with Yap promoter luciferase construct and treated with Lep alone or combination of Lep + Vp. ** = *p* < 0.01, *** = *p* < 0.001 as indicated in the graph. (**C**) Expression of AREG, FOXF1 and CCND1 was analyzed by real-time PCR and normalized to 18s. * = *p* < 0.05, ** = *p* < 0.01, *** = *p* < 0.001 between groups as indicated in the graph. (**D**) Luciferase activity in HSCs transiently transfected with Yap promoter luciferase construct and treated with Vp alone, Rosiglitazone (Rosi) alone or the combination of both. *** = *p* < 0.001 vs. relative WT bar; ° = *p* < 0.01 vs. relative WT Ctrl.

Since leptin (Lep) has also been implicated in controlling the HH pathway in HSCs in addition to TGF-β [17], we hypothesized that leptin could also amplify HH-mediated gene expression in HSCs with the *PNPLA3* variant. First, we observed higher mRNA levels of PNPLA3, TIMP-1 and GLI-2 in I148M HSCs, compared to WT HSCs incubated with recombinant leptin (Figure 5A). These data were in line with the luciferase activity of Yap-induced luciferase activity measured in HSCs treated with Lep (Figure 5B). Interestingly, Yap-specific inhibitor Vp strongly reduced Yap response element transactivation by leptin in both WT and I148M HSCs (*p* < 0.01, Figure 5B) and down-regulated the expression of markers of HH and Yap signaling counteracting the leptin effects (Figure 5C, Figure S1).

Previous reports indicated that down-regulation of PPARγ is essential to the metabolic switch mediated by HH towards anaerobic glycolysis that maintains myofibroblastic features in HSCs [14]. In line also with our prior observations [22], we aimed to test whether the use of the PPARγ synthetic agonist Rosiglitazone (Rosi) was able to counteract Yap signaling. By measuring luciferase Yap transcriptional activity, we found not only that Rosi alone was able to strongly reduce it, but the combination of Rosi and Vp resulted in more than 3-fold down-regulation (Figure 5D). Based on these data, we can conclude that HSCs carrying I148M display higher HH and Yap signaling, resulting in enhanced anaerobic glycolysis and synthesis of downstream targets that contribute to maintain a higher fibrogenic phenotype (AREG, FOXF1, Vimentin). Notably, TGF-β and leptin exacerbate the synthesis of HH/Yap-related pro-fibrotic genes, which can be highly counteracted by the use of the specific Yap inhibitor Vp and PPARγ synthetic agonist Rosiglitazone.

## 3. Materials and Methods

### 3.1. Human HSC Isolation and Cell Culture

Primary HSCs were isolated from donor livers unsuitable for transplantation or liver resections for metastasis of colon-rectal cancer, as described elsewhere [21,22]. HSCs were seeded on uncoated plastic dishes and cultivated with Iscove’s Modified Dulbecco’s Medium (EuroClone, Milan, Italy) supplemented with 20% fetal bovine serum (FBS), 1% glutamine 200 mM, sodium pyruvate 100 mM, non-essential amino acid solution 100× and antibiotic antimycotic solution 100× (Gibco Life Technologies, Carlsbad, CA, USA). Primary HSCs between passages one and eight were used for this study. For comparison, primary WT and I148M *PNPLA3* HSCs were used at the same passage. The genotype of each isolated HSC line was analyzed by real-time polymerase chain reaction (RT-PCR) for the I148M single-nucleotide polymorphism, as performed routinely in our clinical research center. Only homozygote genotypes (*n* = 3) were used in this study (C/C as WT and G/G as I148M). Stably overexpressing WT and I148M LX-2 were generated in our laboratory as described previously [21,22] and cultured with Dulbecco’s modified Eagle’s medium (DMEM, Thermo Fisher scientific, Vienna, Austria) supplemented with 5% FBS and 1% penicillin/streptomycin solution (EuroClone, Milan, Italy). To verify TGF-β-mediated increase in mRNA transcripts, the inhibitor of RNA polymerase II Actinomycin D (Sigma-Aldrich, Vienna, Austria) was used on cells 1h prior to cytokine treatment.

Where indicated in the graphs or in figure legends, cells were deprived for 24 h of FBS and exposed to 5 μM of Yap inhibitor Verteporfin ([Vp]; Sigma-Aldrich) prior to stimulation with 5 ng/mL of TGF-β (Prospec) or with 100 ng/mL of leptin ([Lep]; from PeproTech, Vienna, Austria) or with 10 μM of PPARγ agonist rosiglitazone ([Rosi]; Sigma -Aldrich, Vienna, Austria).

### 3.2. Metabolic Characterization

Analysis of OCR and ECAR was performed using the XF24 Flux Analyzer (Seahorse Bioscience, Copenhagen, Denmark). In brief, LX-2 stably overexpressing either the wild type or I148M *PNPLA3* isoform was seeded into XF 24-well cell culture microplates and allowed to recover for 24h, in culture medium. A final volume of 450 mL of buffer-free Assay Medium (Seahorse Bioscience, Copenhagen, Denmark) was added to each well prior to the experimental protocol. Cells were then transferred to a CO_2_-free incubator and maintained at 37 °C for 1h before starting the assay. Following instrument calibration, cells were transferred to the XF24 Flux Analyzer to record cellular oxygen consumption rates. The measurement protocol consisted of 3 min mixture, 2 min waiting and 3 min ECAR measurement times. At the end of the assay, the medium was carefully aspirated, and a standard protein assay was performed to correct for cell count [26].

### 3.3. Luciferase Assay

The luciferase assay was performed as previously reported [20,21,22]. Briefly, WT- and I148M-overexpressing LX-2 cells were seeded in a 24-well plate and transiently transfected with 0.3 µg/well of the Yap/Taz luciferase reporter, using Fugene transfection reagent (Promega, Madison, WI, USA) in sterile Opti-MEM for 12 h, as previously described [21,22]. After 24 h, the medium was replaced with DMEM glucose-free and cells were treated as previously described for an additional 24 h. Cells were lysed using a solution (4% Triton-X100, Glycyl-Glycine 100 mM, MgSO_4_ 100 mM, EGTA 250 mM) for 1 h at room temperature on a shaker platform. The lysates were then combined with the substrate solution (Luciferin 2.5 mM and ATP 20 mM, Sigma-Aldrich) and analyzed with a luminometer (Lumat LB9507; HVD life science, Vienna, Austria) [27]. The Yap/Taz luciferase reporter, a 8xGTIIC-luciferase construct containing the Yap/Taz-responsive synthetic promoter driving luciferase expression, was a gift from Stefano Piccolo (Addgene plasmid #34615; http://n2t.net/addgene:34615; RRID:Addgene_34615) [13].

### 3.4. Flow Cytometry

LX-2 stably overexpressing WT and I148M *PNPLA3* was previously treated with either Verteporfin, TGF-β or Leptin for indicated time points, trypsinized, permeabilized with Triton X-100 and incubated with a 1:500 dilution of the polyclonal rabbit anti human Yap and p-Yap antibody (Cell Signaling, Danvers, MA, USA) for 1 h, on a shaker platform. Afterwards, Alexa Fluor 594 goat anti rabbit IgG was incubated for 1 h at room temperature on a shaker, DAPI was added to each tube for 10 min before flow cytometric analysis. Flow cytometry was performed with BD FACSCanto™ II and BD FACSDiva™ software (Becton Dickinson New Jersey, Franklin Lakes NJ, USA).

### 3.5. Statistics

Data are presented as mean values ± standard deviation (SD). Statistical analysis was performed using GraphPad Prism (La Jolla, CA, USA). For head to head comparison between two groups, we applied the unpaired Mann–Whitney test. When multiple groups were compared, we performed a two-way analysis of their variance. *p* < 0.05 was considered statistically significant.

## 4. Discussion

A main finding of our current work is that, for the first time, I148M-activated myofibroblasts display pronounced activation of the HH pathway and of its downstream effector, Yap. The latter results in boosted anaerobic glycolysis and enhanced expression of fibrogenic markers which contribute to maintain a myofibroblastic phenotype in HSCs with the I148M variant, compared to WT *PNPLA3* HSCs. Notably, this metabolic reprogramming known as “the Warburg effect” is controlled by HH and it is a key metabolic switch to sustain the fibrogenic activities of these highly proliferative cells [14]. In addition, it was also established that chronic cholesterol elevation in liver leads to TAZ activation in hepatocytes, by blocking its proteasomal degradation [28]. TAZ is a transcription factor activating in hepatocytes the Hedgehog signaling pathway [28]. Therefore, it is tempting to speculate that cholesterol accumulation in hepatocytes may lead to increase Hedgehog signaling and secretion of Indian Hedgehog, activating HSCs expressing the *PNPLA3* I148M variant prone to fibrosis, with cholesterol accumulation due to dysfunctional PPARγ and LXR signaling [21,22]. This cholesterol accumulation in *PNPLA3* I148M HSCs might make them more amenable to Hedgehog signaling in an autocrine manner.

Currently, no approved therapies for the treatment of liver fibrosis exist. A better understanding of the factors regulating hepatic stellate cells (HSCs) activation and liver fibrosis will hopefully facilitate the development of urgently needed novel anti-fibrotic therapies in NASH and other CLD [1]. The genetic I148M variant of the gene coding for *PNPLA3* has been strongly and widely correlated with a higher risk of developing severe hepatic fibrosis in patients with NAFLD/NASH [19] and other forms of liver diseases such as alcoholic hepatitis, viral hepatitis B and C and liver cancer [19]. Previous data demonstrated that during NASH, PNPLA3 tissue expression increases, from mild to severe fibrosis, and correlates with fibrosis stage and α-SMA hepatic tissue expression, independently from the *PNPLA3* genotype [20]. In addition, HSCs carrying I148M *PNPLA3* show enhanced release of inflammatory cytokines, increased migration and proliferation rates and altered PPARγ and LXR signaling pathways resulting in disturbed lipid and cholesterol metabolism [21,22].

Herein, we also report that short-time exposure to TGF-β quickly induces PNPLA3 transcript and protein accumulation in HSCs, confirming previous observations by another group [29]. Interestingly, this is accompanied by fast transcription of the lipogenic gene FASN, while longer time points stimulate the activation of the energy sensor AMPK (p-AMPK) and proportional induction of CPT-1. This change concomitant to the decrease in PNPLA3 transcription may be of key interest, since activated hepatic AMPK (p-AMPK) suppresses de novo lipogenesis through inactivation of acetyl-CoA carboxylase (ACC1 and ACC2) and subsequently reduces the intracellular content of malonyl-CoA, the potent inhibitor of mitochondrial fatty acids oxidation by blocking CPT-1 activity [30]. These data become even more interesting when we compare the levels of activated AMPK between WT and I148M HSCs. Indeed, a significant reduction in the levels of p-AMPK in HSCs carrying the *PNPLA3* genetic variant supports our analysis of the extracellular acidification rate (ECAR), indicating that these cells prefer to boost the consumption of glucose in anaerobic conditions, even though less productively in terms of ATP, rather than relying on mitochondrial respiration. Since our metabolic analysis previously showed that HSCs carrying the I148M variant display both reduced de novo lipogenic gene expression and elevated free and total cholesterol levels [21], these new findings here reported on p-AMPK and anaerobic glycolysis in I148M HSCs represent a step forward necessary to better characterize the metabolic profile of these cells and further propose new tools for their inhibition or reversal.

As previously described by Chen et al. [14] the metabolic switch that leads to lactate accumulation in HSCs is controlled by the HH pathway. In healthy liver, the production of HH ligands is low, while the HH antagonist Hedgehog-interacting protein (HHIP) is high. Our data demonstrate that primary HSCs with the I148M *PNPLA3* have low expression of the HH antagonist (HHIP), while they displayed high levels of the HH agonist Indian Hedgehog (HIP) and CyclinD1 (CCND1, a HH target gene), suggesting an activation of the HH pathway. Remarkably, during trans-differentiation into activated myofibroblasts, HSCs dramatically reduce PPARγ signaling, with a parallel strong synthesis and autocrine release of HH ligands, when activated HSCs treated with synthetic inhibitors of the HH pathway recovered PPARγ activity and lost their myofibroblastic features [14]. This is important to consider, since HSCs with the *PNPLA3* variant show specifically diminished PPARγ signaling [22]. Yes-associated protein 1 (Yap) is a morphogenic signaling protein relatively inactive in healthy liver, but dramatically activated in HSCs during liver injury. Remarkably, Yap controls HSCs activation while the HH pathway regulates Yap signaling, and inhibition of this pathway prevented the transformation of quiescent HSCs into active and proliferative myofibroblasts [24,25,31]. Moreover, in some cells, glycolysis is necessary to switch on the Yap pathway, suggesting that morphogens may modulate metabolism to induce cell fate changes that are required for adult tissues to survive and recover from injury. Furthermore, a positive correlation between glucose transporter GLUT3 and Yap expressions was found in human samples of hepatocellular carcinoma (HCC), along with the discovery of Yap phosphorylation (therefore its inhibition) mediated by activated AMPK, p-AMPK [32]. With this reported evidence taken into account, our observations emphasize the intimate association between enhanced Yap signaling, lower activation of AMPK consequently to boosted glycolysis and the presence of the *PNPLA3* genetic variant in HSCs [22]. Given that phosphorylated Yap (p-Yap) cannot translocate into the nucleus to regulate Yap targets gene expression, the reduced amount of p-Yap here reported at baseline and after TGF-β and leptin supports our finding of increased Yap signaling in HSCs with the *PNPLA3* genetic variant.

In addition to HH, a variety of mechanical signals such as cell–cell interactions and changes in ECM stiffness can activate Yap signaling. Mechanic transduction-induced signals mediated by Yap have been shown to promote generation of cancer-associated fibroblasts (CAFs) [12], and therefore the Yap pathway is one of the main players involved in the regulation of contact inhibition and epithelial to EMT. From this perspective, liver regeneration studies highlighted interactions between TGF-β and Yap signaling pathways to stimulate hepatocytes to undergo an EMT-like response necessary for them to grow in a TGF-β-enriched microenvironment and regenerate injured livers [33]. In line, our data demonstrated that TGF-β triggered Yap transcriptional activity and downstream targets expression, which can be reverted by the specific Yap inhibitor Verteporfin. In this context, parallel studies in pulmonary vascular biology have reported that ECM stiffness potentiates the Yap–glutaminase axis to promote vessel stiffening and matrix remodeling [34], thus supporting the concept that this might represent a mechanosensitive conserved mechanism necessary to maintain myofibroblastic trans-differentiation in HSCs. To note, glutaminase (GLS) is a rate-limiting mitochondrial component for converting glutamine to glutamate, which is then transformed into α-ketoglutarate to enter the tricarboxylic acid cycle. In line, Du and colleagues elegantly demonstrated that during trans-differentiation into activated myofibroblasts, HSCs increase glutamine metabolism (glutaminolysis) due to up-regulated Yap signaling. Thereby, inhibition of Yap and blockade of glutaminolysis resulted in suppression of myofibroblastic features in HSCs [35]. Consistently, here we report that already at baseline, HSCs carrying the *PNPLA3* variant show higher levels of GLS-2 (liver isoform) compared to WT HSCs, and that Verteporfin can abrogate this difference, thus confirming that high GLS levels are compatible with up-regulated Yap signaling also in the presence of the I148M isoform.

**Figure 6:**
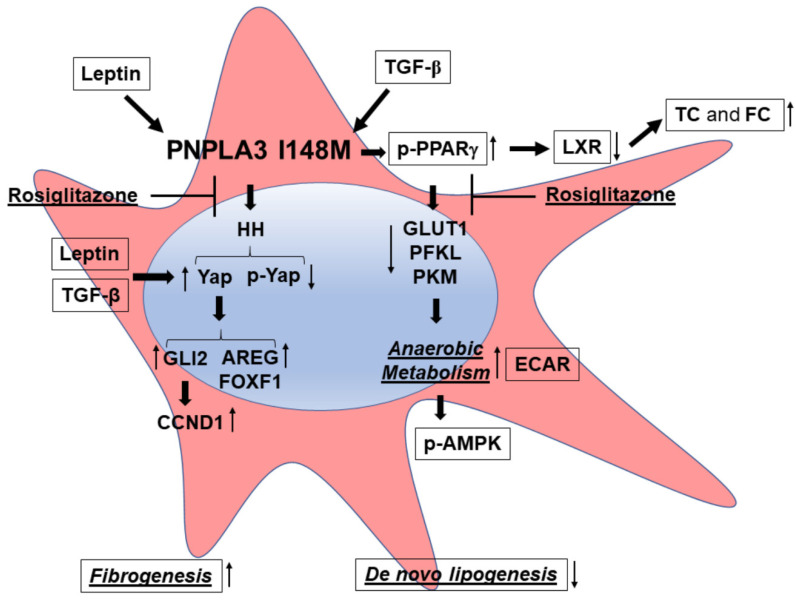
*PNPLA3* I148M promotes activation of HH signaling and its downstream target Yap. Summary scheme representing the major molecular steps characterizing the phenotype of I148M HSCs. The presence of the *PNPLA3* genetic variant leads to disturbed PPARγ and LXR signaling (accumulation of total cholesterol = TC and free cholesterol = FC), resulting in activation of Hedgehog (HH) and Yap signaling and an increase in anaerobic metabolism (higher ECAR and diminished expression of GLUT1, PFKL, PKM). Both TGF-β and Leptin induce expression of Yap, as a target of the HH pathway, and its downstream effectors (Vimentin, FOXF1, AREG, CCND1). Inhibition of Yap by Rosiglitazone leads to decreased Yap transcriptional activity in HSCs carrying *PNPLA3* I148M.

Given the strong contribution of leptin in stimulating the HH pathway to induce and maintain myofibroblast tracts in HSCs [16,17], the present work aimed to test also the contribution of leptin to Hedgehog and Yap signaling in I148M HSCs. For the first time to our knowledge, we here report that leptin up-regulates PNPLA3 expression specifically in cells with the genetic variant, accompanied by induction of pro-fibrotic marker expression, TIMP-1 and the downstream effector of HH, GLI-2. Similar to TGF-β, leptin was able to transactivate the Yap promoter and therefore induce HH and Yap downstream targets, while Verteporfin abrogated these effects. Prior studies strongly highlighted the evidence that leptin interferes with PPARγ signaling by inhibiting PPARγ expression [36,37]. Since our data suggested that a potent PPARγ synthetic agonist, Rosi, partially abolished some fibrogenic characteristics of HSCs with the *PNPLA3* variant [21], we herein provide mechanistic evidence that Rosi counteracts Yap promoter activation and, moreover, the combination with Verteporfin resulted in a potent inhibition of Yap signaling, however to a lower extent in I148M compared to WT HSCs (mechanistic data summarized in Figure 6). Considering that there are no approved drugs for the treatment of nonalcoholic fatty liver disease (NAFLD) or NASH, the present study may reveal some novel opportunities to expand and apply the use of Verteporfin alone or in combination with Rosiglitazone, or other new pan-PPAR agonists displaying a strong PPARγ activity like Lanifibranor [38] for counteracting Yap signaling, which is essential for the activation and maintenance of activated HSCs. Importantly, given that verteporfin is already approved by the US Food and Drug Administration (FDA) for treatment of liver fibrosis, our results should stimulate further mechanistic studies to further explore these observations in animal models for chronic liver injury and fibrosis. Finally, improved understanding of the HSCs phenotype associated with *PNPLA3* I148M should pave the path for novel therapeutic approaches to counteract hepatic fibrogenesis.

## Supplementary Materials

Supplementary materials can be found at https://www.mdpi.com/1422-0067/21/22/8711/s1, Figure S1: Verteporfin inhibits Leptin mediated Hedgehog/Yap target gene expression.

## Abbreviations

AMPK	5′ AMP-activated protein kinase
AREG	amphiregulin
α-SMA	α-smooth muscle actin
CCND1	cyclin D1
COLL1α1	type I collagen
CPT-1	carnitine palmitoyl-transferase 1
ECM	extracellular matrix
FASN	fatty acid synthase
FFA	free fatty acid
FOXF1	forkhead box protein F1
GLS-2	glutaminase 2
GLUT1	glucose transporter 1
GM-CSF	granulocyte-macrophage colony-stimulating factor
HCC	hepatocellular carcinoma
HH	hedgehog
HIP	indian hedgehog
HHIP	hedgehog-interacting protein
HK	hexokinase-2
HSCs	hepatic stellate cells
Lep	leptin
LPK	liver pyruvate kinase
NAFLD	non-alcoholic fatty liver disease
NASH	non-alcoholic steatohepatitis
PFKL	phosphofructokinase liver type
PKM	pyruvate kinase muscle
PNPLA3	patatin-like phospholipase domain-containing protein 3 (C/C = wild type)
PNPLA3 I148M	patatin-like phospholipase domain-containing protein 3 genetic variant I148M (G/G)
TG	triglycerides
Vp	verteporfin
WT	PNPLA3 wild type (C/C)
Yap	yes-associated protein 1
