# Supplementary material for: PNPLA3 I148M Up-Regulates Hedgehog and Yap Signaling in Human Hepatic Stellate Cells
Source: Int J Mol Sci. 2020 Nov 18;21(22):8711. doi: 10.3390/ijms21228711 (PMC7698885; doi:10.3390/ijms21228711)
Supplement: Supplementary file 1 [file ijms-21-08711-s001.pdf]

PNPLA3 I148M Up-Regulates Hedgehog and Yap Signaling in Human Hepatic Stellate Cells

Francesca Virginia Bruschi [1], Matteo Tardelli [1,2], Elisa Einwallner [3], Thierry Claudel [1] and Michael Trauner [1,*]

[1] Hans Popper Laboratory of Molecular Hepatology, Division of Gastroenterology and Hepatology, Department of Internal Medicine III, Medical University of Vienna, Vienna 1090, Austria; francesca_bruschi@yahoo.it (F.V.B.); mat4005@med.cornell.edu (M.T.); thierry.claudel@meduniwien.ac.at (T.C.)

[2] Division of Gastroenterology and Hepatology, Joan and Sanford I. Weill Cornell Department of Medicine, Weill Cornell Medical College, New York, NY 10021, USA

[3] Department of Laboratory Medicine, Center of Translational Research, Medical University of Vienna, Vienna, 1090, Austria; elisa.einwallner@meduniwien.ac.at

* Correspondence: michael.trauner@meduniwien.ac.at; Tel.: +43-140-4004-7410; Fax: +43-14-0400-4735

Table of Contents:

- Supporting Materials and Methods
- Supporting Figure and Figure Legends

Supporting Materials and Methods:

RT-PCR analysis:

RNA was isolated from primary HSCs and LX-2 by NucleoSpin® RNA (MACHEREY-NAGEL GmbH & Co., Germany) according to manufacturer's instructions. One µg of RNA was reverse transcribed using Moloney Murine Leukemia Virus enzyme, 10xbuffer for cDNA synthesis and Deoxynucleotide Mix 10mM (Sigma-Aldrich). Two microliters of diluted cDNA were loaded together with SYBR® Select Master Mix on 96 well-plate to perform RT-PCR analysis (Thermo Fisher Scientific). Forward and reverse primers for each gene of interest were specifically designed and used at 10 pmol/µL (Eurofins Genomics, Germany). Sequences are available upon request.

Supporting Figure and Figure Legend:

Supplementary
Fig.1
Bruschi et al.

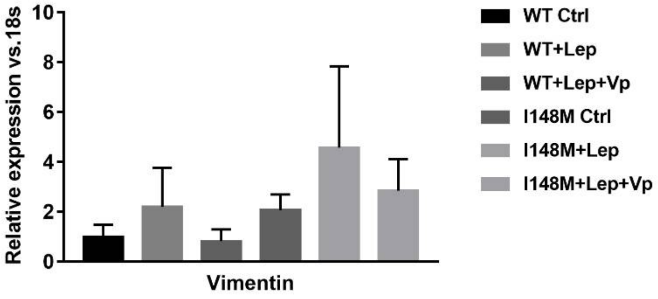

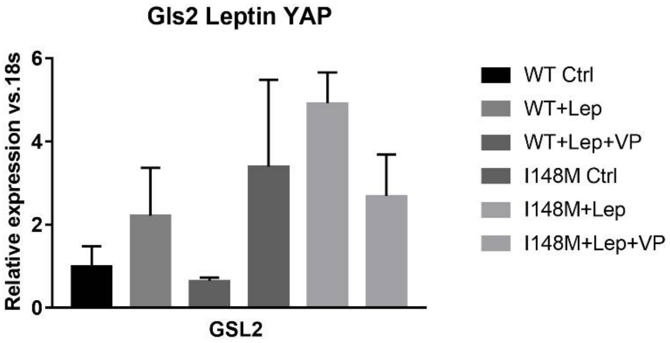

Supp. Fig.1. Verteporfin inhibits Leptin mediated Hedgehog/Yap target gene expression. LX-2 stably overexpressing cells (*n* = 3 each PNPLA3 genotype) obtained and cultivated in vitro, as described in Materials & Methods. Where indicated, cells were stimulated with Vp and Leptin (Lep) respectively for 24h and 1h prior the analysis. Expression of Vimentin and GLS2 analyzed by real-time PCR and normalized to 18s.
